# Pyrene-Chromone Schiff Base Molecules with Tunable Fluorescence: Structure–Property Relationships and Substituent Effects

**DOI:** 10.3390/molecules31061059

**Published:** 2026-03-23

**Authors:** Merve Zurnacı

**Affiliations:** Central Research Laboratory, Kastamonu University, 37200 Kastamonu, Turkey; mzurnaci@kastamonu.edu.tr

**Keywords:** pyrene, chromone, Schiff base, fluorescence, absorption, emission, aggregation

## Abstract

The fluorescence properties of organic molecules are largely determined by molecular architecture, π-conjugation, and electronic substituent effects. In this study, three novel pyrene-chromone Schiff base derivatives were designed and synthesized to investigate substituent-driven modulation of photophysical behavior. The compounds were obtained via condensation of 1-aminopyrene with three different chromone-based aldehydes and fully characterized by FT-IR, ^1^H-NMR, and mass spectrometry. The molecular design involves a donor-π-acceptor architecture: pyrene donates electrons, while the chromene moiety accepts them, enabling charge transfer upon excitation. UV-Vis and fluorescence spectroscopy revealed intense absorption in the 430–440 nm range and tunable emission in the 540–565 nm region, corresponding to large Stokes shifts (107–125 nm). Substituent effects significantly influenced optical band gaps and emission intensities, with the nitro-substituted derivative exhibiting a reduced band gap and pronounced fluorescence quenching due to enhanced intramolecular charge transfer. Concentration-dependent absorption studies demonstrated linear Beer–Lambert behavior, indicating the absence of aggregation within the investigated range. These results establish clear structure–property relationships in pyrene-chromene Schiff bases and highlight their potential as promising candidates for optoelectronic and fluorescence-based sensing applications.

## 1. Introduction

Schiff bases have attracted considerable attention due to their versatile coordination ability, facile synthesis, and broad range of applications in biological, electrochemical, and photophysical fields [1,2,3,4,5]. In particular, fluorescence-active Schiff base derivatives have emerged as promising functional materials for chemical sensing, organic light-emitting diodes (OLEDs), organic field-effect transistors (OFETs), and dye-sensitized solar cells (DSSCs) [6,7,8,9]. Their structural flexibility allows tuning of electronic properties through molecular design, making Schiff bases attractive platforms for developing luminophores with tailored optical responses [10,11,12,13]. Despite extensive reports on Schiff base luminophores, a unified framework correlating substituent effects with photophysical behavior has not yet been fully established [14,15,16]. In this context, donor-π-acceptor (D-π-A) architectures based on Schiff bases are particularly promising for such research.

**Figure 1:**
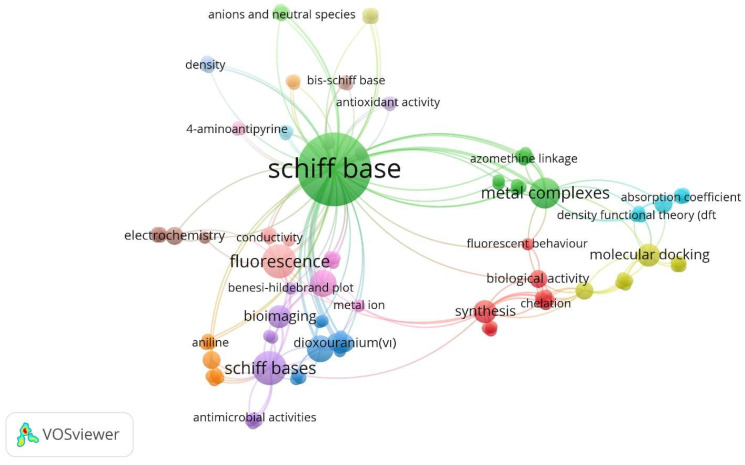
Bibliometric network of research topics related to Schiff base systems (VOSviewer, version 1.6.20).

The growing interest in Schiff base derivatives has resulted in a substantial body of literature spanning diverse research domains. Nevertheless, the thematic distribution and research directions within this field continue to evolve. To obtain an overview of current trends, a VOSviewer (version 1.6.20)-based bibliometric analysis was performed, and the results are presented in Figure 1. The resulting map illustrates the major thematic clusters. It highlights the positioning of Schiff base systems within the broader research landscape, thereby providing contextual support for the motivation and scope of the present study.

Among the various fluorophore scaffolds incorporated into Schiff bases, polycyclic aromatic hydrocarbons, such as pyrene, naphthalene, phenanthrene, and anthracene, exhibit strong fluorescence originating from their extended π-conjugated frameworks [17,18]. In particular, pyrene has attracted considerable attention in optical and electronic applications owing to its high fluorescence quantum yield, long excited-state lifetime, chemical stability, and large planar π-surface, which facilitates efficient electronic delocalization [19,20,21]. These features render pyrene a versatile building block for the development of highly emissive organic materials [22,23,24,25].

Previous studies have demonstrated that pyrene-based Schiff base derivatives exhibit diverse photophysical behaviours depending on molecular framework and substitution pattern [23,24,26,27]. Reported systems have been explored as fluorescent probes for metal ions [28], pigments [29], antimicrobial agents [24], and optoelectronic materials [27], with emissions spanning the blue to green-yellow region. In many cases, modulation of the fluorescence wavelength and intensity has been associated with intramolecular charge transfer (ICT) [30], aggregation-induced effects [31], or photoinduced electron transfer processes [26]. Collectively, these observations demonstrate that pyrene–Schiff base platforms constitute a flexible molecular scaffold for the construction of tunable fluorophores [27,32,33].

Pyrene-based Schiff bases have been widely investigated for their fluorescence and sensing properties [34,35,36,37]. Similarly, chromone-derived Schiff bases have been reported to possess various optical and biological activities [38,39,40]. However, studies integrating both pyrene and chromone moieties within a single conjugated Schiff base framework remain relatively limited. For example, Jyoti Chourasia et al. (2023) reported a pyrene–chromone imine-based system as a fluorogenic probe for the detection of organophosphate nerve gas mimics [41]. In this study, however, the molecular structure was specifically designed for chemosensing applications and employed a different molecular architecture derived from the pyrene-chromone skeleton. The derivatives examined in the present work differ in both their structural frameworks and substitution patterns. To the best of our knowledge, structurally comparable derivatives with similar substitution patterns have not been systematically investigated. In particular, systematic studies exploring how structural modifications in pyrene–chromone Schiff base derivatives influence the electronic interaction between these two π-active units and key photophysical parameters, such as the optical band gap, Stokes shift, and aggregation behavior, remain limited. Therefore, integrating pyrene and chromone units into newly designed Schiff base derivatives and systematically investigating their structure–photophysical property relationships provides additional insights into the design of tunable fluorescent materials.

In this study, the molecular architecture of the synthesized compounds was designed based on a donor-π-acceptor (D-π-A) framework. In this configuration, the electron-rich pyrene unit functions as the donor, while the chromone moiety acts as the electron acceptor, connected through a conjugated imine (C=N) bridge. Such an arrangement is expected to promote electronic communication across the π-conjugated system and facilitate intramolecular charge transfer (ICT) upon excitation. In this design, photoexcitation is expected to redistribute electron density from the pyrene donor to the chromone acceptor, resulting in an ICT-dominated excited state. This behavior is commonly observed in donor–acceptor fluorophores, where the electronic coupling between the donor and acceptor moieties determines the optical band gap and emission properties.

In this context, the present study reports the design and synthesis of three novel pyrene-chromone Schiff base derivatives and a comprehensive evaluation of their structural and photophysical properties. Particular emphasis is placed on elucidating the influence of different substituent groups on absorption behaviour, emission characteristics, optical band gaps, and aggregation tendencies. The obtained findings aim to provide clear structure–property correlations and to demonstrate the potential of these materials for optoelectronic and fluorescence-based sensing applications.

## 2. Results and Discussion

### 2.1. Structural Characterization

The target Schiff base derivatives were successfully synthesized and fully characterized by FT-IR, ^1^H-NMR, and LC–MS/MS techniques. The obtained spectroscopic and analytical data are consistent with the proposed molecular structures. The experimental data for the synthesized compounds are summarized in Table 1. All compounds were isolated in good yields (>78%). The FT-IR, ^1^H NMR, and mass spectral data are discussed below. The ^1^H NMR spectra of all compounds are provided in Figure S1 in the Supplementary File.

#### 2.1.1. FT-IR Spectra

**Figure 2:**
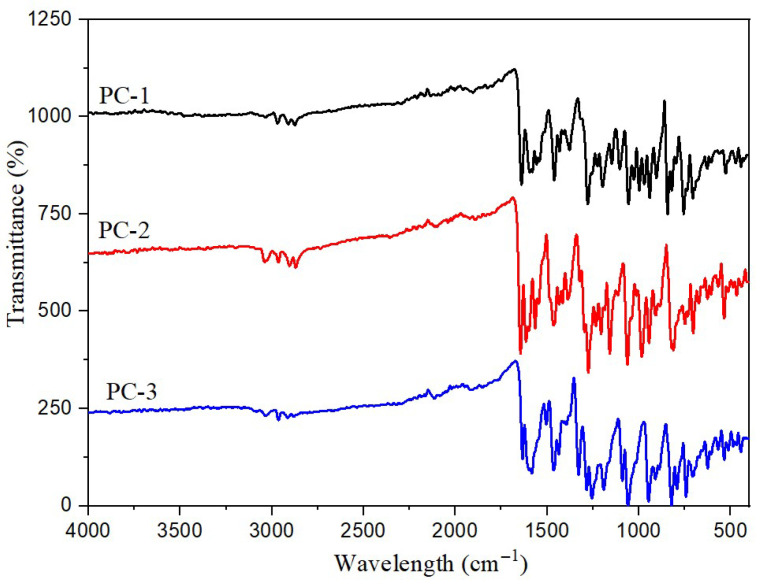
FTIR spectra of PC-1, PC-2, and PC-3.

The FT-IR spectra of the synthesized PC-1, PC-2, and PC-3 compounds are given in Figure 2. Examining the FT-IR spectrum of PC-1, it is observed that the small and medium intensity peaks seen at 3036.74 cm^−1^ and 2969.80 cm^−1^ are due to aromatic C–H stretching, while the peaks seen at 2910.55 cm^−1^ and 2875.11 cm^−1^ are due to aliphatic C–H stretching absorption. The peak seen at 1639.28 cm^−1^ corresponds to C=O stretching vibrations. The peak assigned to the C=N stretching mode of the Schiff base is observed at 1593.23 cm^−1^. The stretching vibration belonging to the C-O group is observed at 1277.79 cm^−1^. Examining the FT-IR spectrum of PC-2, it is observed that the small and medium intensity peaks seen at 3039.65 cm^−1^ and 2964.97 cm^−1^ are aromatic C–H stretching peaks, while the peaks seen at 2903.99 cm^−1^ and 2870.04 cm^−1^ are aliphatic C–H stretching absorption peaks. The peak seen at 1644.38 cm^−1^ corresponds to C=O stretching vibrations. The peak assigned to the C=N stretching mode of the Schiff base is observed at 1614.53 cm^−1^. The stretching vibration belonging to the C–O group was observed at 1274.91 cm^−1^. When the FT-IR spectrum of PC-3 is examined, the small-to-medium intensity peaks observed at 3083.63 cm^−1^, 3035.37 cm^−1^, and 2964.29 cm^−1^ appear to be aromatic C-H stretching absorptions. In contrast, the peaks at 2913.21 cm^−1^ and 2880.26 cm^−1^ are aliphatic C-H stretching absorptions. The peak observed at 1633.90 cm^−1^ corresponds to C=O stretching vibrations. The peak assigned to the C=N stretching mode of the Schiff base is observed at 1582.77 cm^−1^. Two peaks at 1503.73 and 1394.77 cm^−1^ are seen to belong to the NO_2_ group. The stretching vibration belonging to the C–O group was observed at 1284.10 cm^−1^.

The presence of a C=N stretching band in all compounds clearly confirms the formation of a Schiff base. The substituent difference observed in PC-3 (–NO_2_) is clearly distinguishable by FT-IR. The similar ranges of the C=O and C–O bands in all compounds indicate that the basic skeletal structure is conserved. Small shifts in the C=N band suggest that electronic effects can alter substituents. Compared to PC-1 (1593.23 cm^−1^), methyl-substituted PC-2 shows a significant upward shift to 1614.53 cm^−1^. The electron-donating inductive effect of the –CH_3_ group increases the bond strength of the imine bond. In contrast, nitro-substituted PC-3 shows a downward shift to 1582.77 cm^−1^ due to the strong electron-withdrawing nature of the –NO_2_ group (Table 2). –NO_2_ reduces the electron density in the C=N bond and weakens the strength constant. These systematic shifts provide strong spectroscopic evidence for the electronic effect of substituents on imine functionality and are consistent with π-electron delocalization.

#### 2.1.2. ^1^H-NMR Spectra

The ^1^H-NMR spectra of PC-1, PC-2, and PC-3 were recorded in DMSO-d_6_, and the spectra are provided in Figure S1 in the Supplementary File. The chemical shifts and multiplicities of the observed resonances are summarized in Table 3. Overall, the spectra are consistent with the proposed pyrene–chromone Schiff base structures and reflect the influence of substituents located on the chromone ring.

In all three compounds, a singlet observed at approximately 6.0–6.3 ppm (H2) corresponds to the isolated proton located on the heterocyclic chromone framework. The nearly identical chemical shift of this signal across the series indicates that this position is only weakly affected by substitution on the aromatic ring. Similarly, the resonance assigned to H1 appears as a singlet at 8.28–8.32 ppm, which is consistent with the deshielded environment adjacent to the imine (C=N) linkage within the conjugated system.

The aromatic protons located on the chromone ring (H3–H6) appear in the region between 7.0 and 8.0 ppm and display coupling patterns characteristic of adjacent aromatic protons. In PC-1, these signals were relatively well resolved and exhibited a sequential coupling pattern. The signals assigned to H3 (7.96 ppm, d), H4 (7.17 ppm, t), H5 (7.55 ppm, t), and H6 (7.11 ppm, d) form a consistent aromatic spin system within the chromone ring. The corresponding COSY spectrum (Figure S2 in the Supplementary File) showed correlations between H3–H4, H4–H5, and H5–H6, confirming the expected connectivity between these adjacent protons and supporting the proposed assignments. In PC-2, the substitution of the chromone ring with a methyl group results in the disappearance of the H4 signal observed in PC-1. The methyl substituent appears as a singlet at 2.33 ppm (3H), which is characteristic of an aromatic methyl group. The remaining chromone protons, H5 (7.35 ppm, d) and H6 (7.00 ppm, d), show an orthocoupling pattern typical for adjacent aromatic protons. This assignment is supported by the COSY spectrum of PC-2 (Figure S3 in the Supplementary File), which shows a correlation between H5 and H6, confirming their coupling relationship within the chromone ring. In PC-3, the chromone ring bears a nitro substituent that exerts a strong electron-withdrawing effect. Consequently, several aromatic signals appear slightly downfield relative to those observed in PC-1 and PC-2. In PC-3, the signals corresponding to H3 and H5 appear in the same aromatic region at approximately 8.72 ppm and overlap to form a multiplet. The signal assigned to H6 (7.37 ppm, d) remains sufficiently resolved to display an ortho-coupling pattern typical of adjacent aromatic protons. The COSY spectrum of PC-3 (Figure S4 in the Supplementary File) shows a correlation between H5 and H6, which supports the proposed assignments within the chromone ring, despite partial signal overlap.

The protons belonging to the pyrene aromatic core (H7–H15) appear collectively in the 8.6–8.0 ppm region in all three compounds. In this region, the spectra exhibit extensive signal overlap arising from the large number of chemically similar aromatic protons within the fused polycyclic pyrene framework. Such spectral congestion is typical for polyaromatic systems and often results in complex multiplet patterns. This behavior is commonly observed for polycyclic aromatic hydrocarbons such as pyrene due to extensive magnetic anisotropy and second-order coupling effects. Therefore, these resonances are conservatively reported as multiplets in Table 3. Consistent cross-peaks observed in this region of the COSY spectra further indicate the presence of multiple coupled aromatic protons within the pyrene framework. In summary, the observed chemical shift trends, substituent-dependent spectral changes in the chromone ring, and correlations detected in the COSY spectra provide consistent spectroscopic evidence for the proposed structures of the synthesized pyrene-chromone Schiff base derivatives.

#### 2.1.3. Mass Spectra

**Figure 3:**
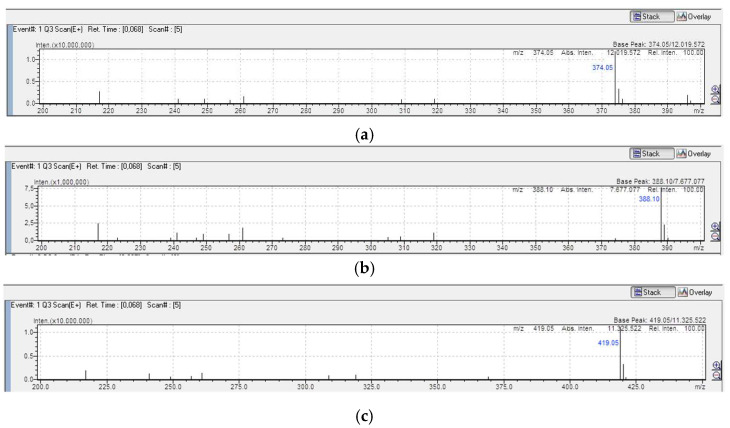
Mass spectra of (**a**) PC-1, (**b**) PC-2, and (**c**) PC-3.

Mass spectrometry measurements of the synthesized PC-1, PC-2, and PC-3 compounds were performed in positive ion scanning mode, and the resulting spectra are given in Figure 3, respectively. It was determined that the experimentally determined molecular weights for all compounds agreed with the theoretically calculated values. The calculated molecular weight for PC-1 is 373.40 g/mol, and the [M + H]^+^ peak observed at 374.05 m/z in the LC-MS/MS spectrum confirms this value. Similarly, the calculated molecular weight for PC-2 was 387.42 g/mol, and a peak corresponding to the [M + H]^+^ ion at 388.10 m/z was observed in the experimental spectrum. In PC-3, the theoretical molecular weight is 418.40 g/mol, which is consistent with the [M + H]^+^ peak observed at 419.05 m/z in LC-MS/MS analysis. These results confirm the proposed molecular structures and molecular weights of the synthesized PC-1, PC-2, and PC-3 with high accuracy.

### 2.2. Photophysical Characterization of Schiff Bases

**Figure 4:**
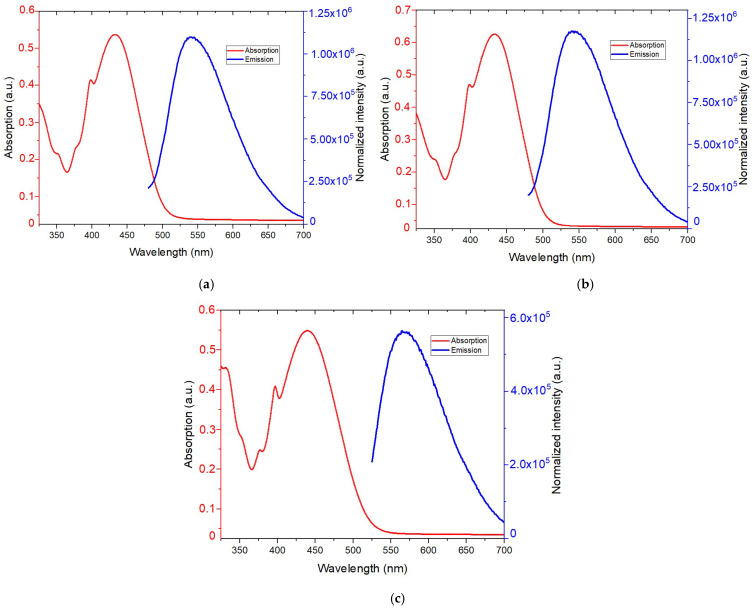
UV-Vis absorption-fluorescence spectra of (**a**) PC-1 (**b**) PC-2 (**c**) PC-3.

Absorption and fluorescence spectra were used to determine the photophysical properties of the synthesized Schiff bases. All measurements were performed in DMSO, a polar aprotic solvent in which the compounds exhibited good solubility. Figure 4 shows the absorption and emission spectra of PC-1 (a), PC-2 (b), and PC-3 (c) in DMSO, respectively. The absorption maximum is observed around 430–450 nm, while the emission maximum appears in the 540–560 nm range, indicating a significant Stokes shift.

**Figure 5:**
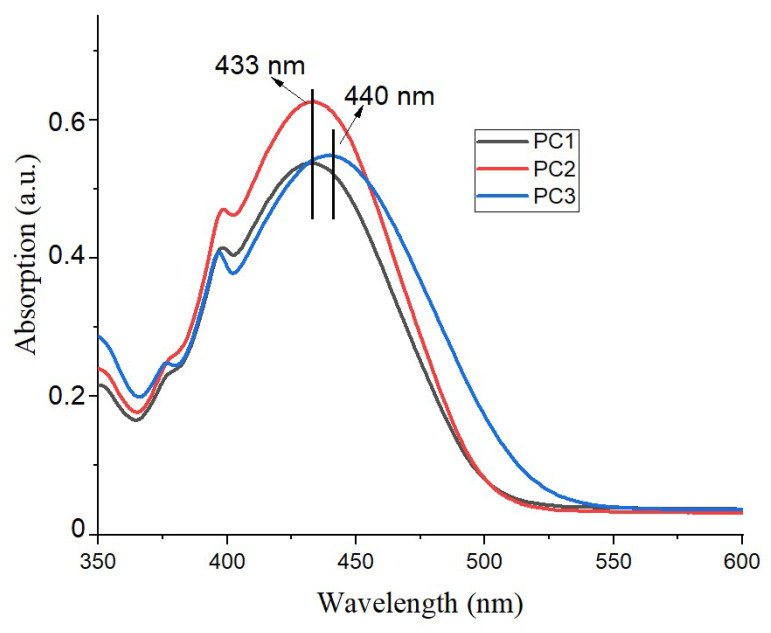
UV absorption graph of compounds.

In the UV-Vis spectra (Figure 5 and Table 4), all compounds exhibited broad π–π* transitions in the 400–460 nm range, attributable to the pyrene-chromone Schiff base framework. Compared to the unsubstituted reference PC-1 (λ_max_ = 432 nm), the methyl-substituted PC-2 showed a slight bathochromic shift (λ_max_ = 433 nm), while the nitro-substituted PC-3 showed a more pronounced red shift (λ_max_ = 440 nm). This trend is consistent with enhanced intramolecular charge transfer (ICT) in PC-3, driven by the strong electron-withdrawing nature of the –NO_2_ group, which stabilizes the excited state and shifts the absorption to longer wavelengths. Electron-accepting substituents such as nitro groups are well known to promote charge-transfer interactions in donor–π–acceptor systems by lowering the LUMO energy and stabilizing the excited ICT state, leading to bathochromic shifts in both absorption and emission spectra [42,43]. In addition, the broader band shape and the extended long-wavelength observed for PC-3 further support an increased charge-transfer character. Such spectral data is commonly associated with ICT transitions because they involve multiple vibronic states [44].

Optical band gaps (E_g_, _opt_) were determined from the absorption maximum wavelength (λ_max_, _abs_) using Equation (1). While PC-1 and PC-2 exhibited similar values (2.87 and 2.86 eV, respectively), PC-3 showed a slightly lower band gap (2.82 eV), consistent with the narrowing of the effective HOMO-LUMO separation by the addition of the electron-withdrawing –NO_2_ group. Notably, the compound with the smallest optical band gap (PC-3) also showed the lowest emission intensity. This can be attributed to a greater contribution of non-radiative decay pathways in the system.

(1) $$E_{g}(eV)=1240/\lambda (nm)$$

**Figure 6:**
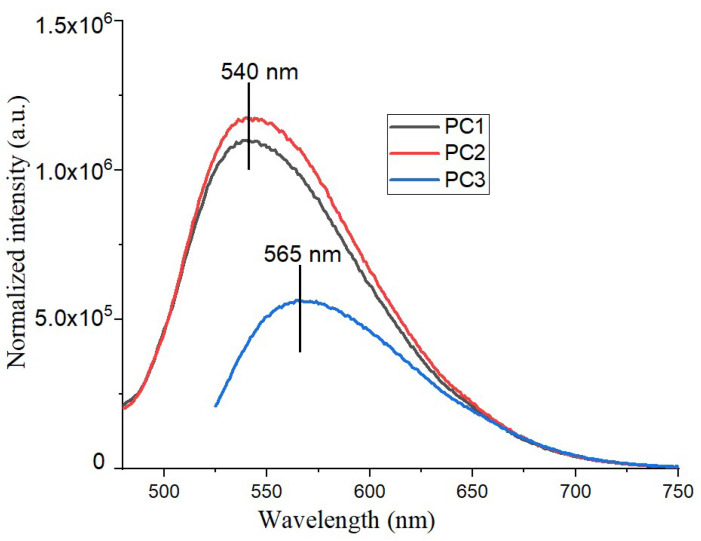
Fluorescence emission graph of compounds.

The fluorescence spectra exhibited emission bands at 540–565 nm, which are significantly red-shifted relative to the characteristic monomer emission of pyrene, typically observed at 370–400 nm [45]. In addition, the emission bands are relatively broad and lack the structured vibronic features commonly associated with pyrene monomer emission. Such red-shifted, broadened fluorescence profiles are frequently observed in donor-π-acceptor fluorophores that exhibit charge-transfer excited states. Given that the molecular architecture forms a donor-π-acceptor framework with pyrene as the electron-rich donor and chromone as the electron-accepting unit, these spectral characteristics are consistent with intramolecular charge-transfer interactions within the conjugated system, a well-established photophysical feature of donor–acceptor pyrene-based chromophores reported in previous studies [46,47]. Fluorescence emission spectra (Figure 6 and Table 5) reveal substituent-dependent modulation of emission intensity and wavelength. PC-1 and PC-2 emit at 540 nm with high intensity (1.17 × 10^6^ and 1.09 × 10^6^ cps, respectively), whereas PC-3 exhibited a red-shifted emission at 565 nm but substantially lower intensity (5.59 × 10^5^ cps), resulting in a larger Stokes shift (125 nm). The incorporation of –NO_2_ into the pyrene-chromone skeleton results in a characteristic red-shifted, but quenched, emission profile compared to PC-1 and PC-2. The larger Stokes shift observed for PC-3 relative to PC-1 and PC-2 suggests a greater degree of excited-state relaxation, consistent with enhanced intramolecular charge transfer supported by the strongly electron-attracting–NO_2_ group.

**Figure 7:**
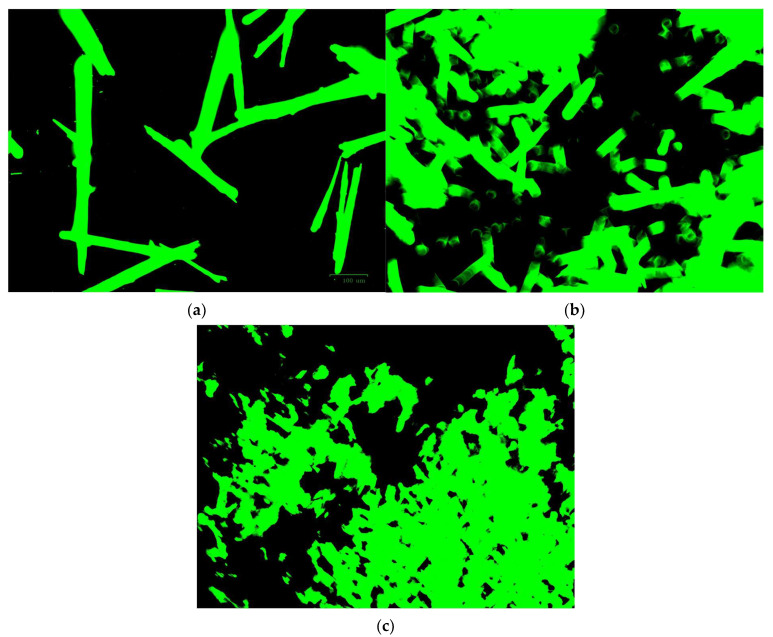
Fluorescence microscopy images of powder mode in green filter: (**a**) PC-1, (**b**) PC-2, and (**c**) PC-3 (green filter, 100 μm).

Solid-state fluorescence was qualitatively visualized by fluorescence microscopy (Figure 7) using a green filter, in which all three compounds showed observable emission in powder form. The microscopy images support the persistence of fluorescence in the solid state and are consistent with the solution-phase emissive behavior.

A comparative summary of the absorption-emission properties and investigated characteristics of some pyrene-based Schiff bases reported in the literature is presented in Table 6. Previously reported pyrene-based Schiff bases typically exhibit absorption maxima in the 347–394 nm range and emission bands in the blue region (410–460 nm). These systems have been predominantly used for metal-ion detection and chemical detector applications. In contrast, the current pyrene-chromone derivatives synthesized in this study exhibit significantly red-shifted emission bands (540–565 nm), indicating a stronger intramolecular charge-transfer character. This different photophysical behavior highlights the effect of the designed donor–acceptor architecture and distinguishes the current system from previously reported systems.

**Figure 8:**
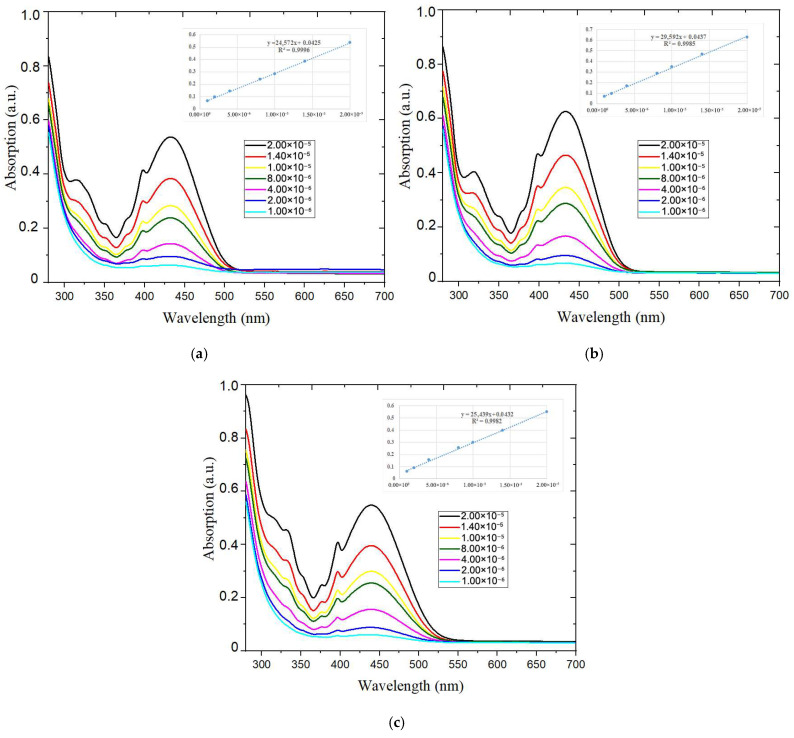
UV-Vis spectrum for aggregation of (**a**) PC-1, (**b**) PC-2, (**c**) PC-3.

The aggregation behavior of the compounds was investigated by concentration-dependent UV-Vis measurements in DMSO in the range of 2.0 × 10^−5^ to 1.0 × 10^−6^ M (Figure 8). Absorbance increased linearly with concentration (R^2^ = 0.998–0.999), and no appreciable changes in λ_max_, band shape, or spectral profile were observed. These findings indicate that the compounds remain predominantly monomeric over the studied range and do not form discernible H- or J-type aggregates under these conditions. This aggregation-free behavior is particularly advantageous for sensing and optoelectronic applications, as it ensures reproducible optical responses that are not complicated by concentration-dependent aggregation phenomena. The absence of aggregation is plausibly attributed to the strong solvation capability of DMSO, which suppresses intermolecular π-π stacking interactions. Collectively, the combination of strong absorption, large Stokes shifts, and aggregation-free spectral behavior in DMSO suggests that these pyrene-chromone Schiff bases are promising candidates for optoelectronic and fluorescence-based sensing applications.

## 3. Materials and Methods

The synthesis procedures employed 1-aminopyrene, 3-formylchromone, 3-formyl-6-methylchromone, 3-formyl-6-nitrochromone (Sigma-Aldrich, St. Louis, MO, USA), and ethanol (Merck, Darmstadt, Germany) as starting materials. All chemicals were of analytical grade (95% or higher) and used without further purification. The melting points of all Schiff bases were measured using a Stuart SMP 30 (Cole-Parmer, Stone, Staffordshire, UK). FT-IR spectra were recorded in the range of 400–4000 cm^−1^ using a Bruker Alpha spectrometer (Bruker Optik GmbH, Ettlingen, Germany). ^1^H-NMR measurements were acquired on an Agilent Premium Compact 600 MHz (14.1 Tesla) NMR spectrometer (Agilent Technologies, Santa Clara, CA, USA). COSY spectra of all compounds were recorded using a Bruker Avance NEO 500 NMR spectrometer (Bruker BioSpin GmbH, Rheinstetten, Germany). Mass spectrometric analyses were performed using a Shimadzu LCMS-MS/8030 Plus system (Shimadzu Corporation, Kyoto, Japan) operated in positive-ion mode. UV-Vis absorption spectra were obtained in the 200–700 nm range using a Shimadzu UV-1700 spectrophotometer (Shimadzu Corporation, Kyoto, Japan), while fluorescence emission measurements were performed on a Horiba FluoroMax-3 spectrofluorometer (Horiba Scientific, Kyoto, Japan). Fluorescence images in powder form were acquired using a BIO-RAD fluorescence microscope (Bio-Rad Laboratories, Hercules, CA, USA) with three fluorescence channels (blue, green, and red) and a digital camera.

### Synthesis of Pyrene-Based Schiff Bases

**Scheme 1:**
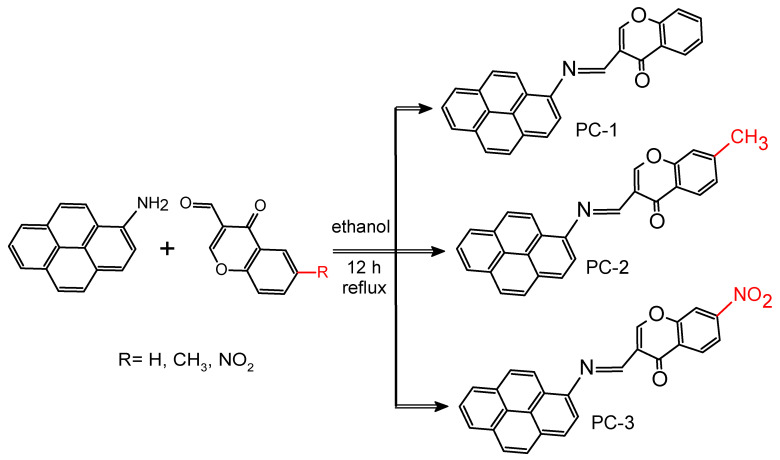
Synthesis of Schiff bases.

The pyrene-based Schiff bases were synthesized via condensation of 1-aminopyrene with chromone-based aldehyde derivatives, according to a literature-reported procedure with minor modifications [21]. In a typical synthesis, 0.5 g of 1-aminopyrene (0.002 mol) was dissolved in 50 mL of ethanol, and a solution of 0.35 g of 3-formylchromone (0.002 mol) in ethanol was added dropwise. Subsequently, glacial acetic acid was introduced dropwise to adjust the pH to 4–5. The reaction mixture was refluxed for 12 h under continuous stirring. After completion, the resulting precipitate was collected by filtration, thoroughly washed with distilled water, and rinsed with diethyl ether to remove residual impurities. The solid products were dried under ambient conditions, and the yields were calculated. The same procedure was applied to other chromone aldehyde derivatives. The synthetic route and chemical structures of the obtained compounds are illustrated in Scheme 1. Additionally, images of the synthesized compounds are shown in Image S1 of the Supplementary Information.

## 4. Conclusions

In summary, this study successfully synthesized three novel pyrene-chromone hybrid Schiff base derivatives and investigated their structural and photophysical properties in detail. FT-IR, ^1^H-NMR, and mass spectrometry analyses confirmed the structures of the synthesized compounds. All spectroscopic results are consistent with the proposed donor-π-acceptor (D-π-A) molecular architecture.

Among the synthesized pyrene-chromone derivative Schiff bases, the PC-3 compound containing a nitro substituent showed the most pronounced structure-fluorescence emission relationship. In the FT-IR spectrum, the shift in the C=N stretching vibration for PC-3 indicates increased delocalization of the imine bond and intramolecular charge transfer due to the strong electron-withdrawing effect of the nitro group. Consistently, PC-3 exhibits a more pronounced bathochromic shift in both UV–Vis absorption and fluorescence emission spectra relative to PC-1 and PC-2. In parallel, the optical band gap of PC-3 was calculated to be 2.82 eV, which is lower than those of PC-1 (2.87 eV) and PC-2 (2.86 eV). This band gap narrowing reflects stabilization of the LUMO level by the nitro substituent and supports increased charge-transfer character in the excited state. In addition, a relative decrease in emission intensity was observed for PC-3, attributed to enhanced non-radiative decay pathways driven by strong electron-attracting and charge-separating effects.

Collectively, the results demonstrate that substituent effects provide an effective strategy for tuning absorption, emission wavelength, emission intensity, and optical band gap in pyrene-chromone Schiff base systems. In particular, the nitro substituent plays a decisive role in determining the electronic structure and fluorescence behavior of these molecules.

These findings establish clear structure–property relationships and highlight pyrene-chromone Schiff bases as promising platforms for the design of tunable luminophores for optoelectronic and fluorescence-based sensing applications.

## Figures and Tables

**Table 1:** Experimental and physical data for the synthesized Schiff base derivatives.

Molecular Structures	Molecular Formula	Molecular Weight	M.P. (°C)	Yield (%)
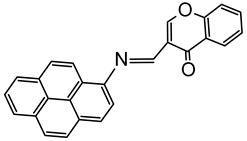 *N-(pyren-1-yl-imino)-3-formyl chromone (PC-1)*	C_26_H_15_NO_2_	373.40	176	81
** 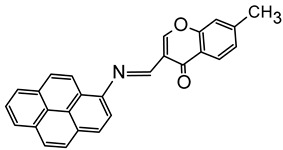 ** *N-(pyren-1-yl-imino)-3-formyl-6-methylchromone (PC-2)*	C_27_H_17_NO_2_	387.42	260	78
** 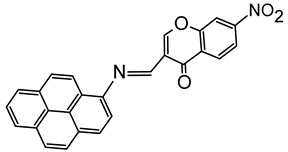 ** *N-(pyren-1-yl-imino)-3-formyl-6-nitrochromone (PC-3)*	C_26_H_14_N_2_O_4_	418.40	>300	79

M.P. = Melting point.

**Table 2:** Substituent-dependent shifts of the imine (C=N) stretching bands in PC-1–PC-3.

Compounds	Substituent	ν (C=N) (cm^−1^)	Δν (Compared to PC-1)
PC-1	–H	1593.23	–
PC-2	–CH_3_	1614.53	+21.30
PC-3	–NO_2_	1582.77	−10.46

**Table 3:** ^1^H-NMR chemical shifts and multiplicities of PC-1-3 recorded in DMSO-d_6_.

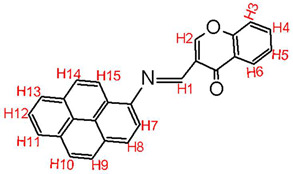		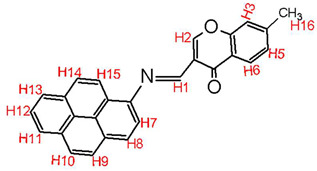	
PC-1		PC-2	
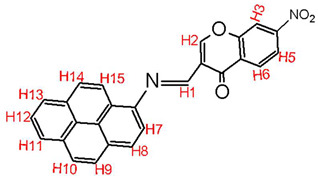			
PC-3			
^1^H-NMR			
	**PC-1 δ (ppm)** Multiplicity (J, Hz) *	**PC-2 δ (ppm)** Multiplicity (J, Hz) *	**PC-3 δ (ppm)** Multiplicity (J, Hz) *
H1	8.28 (s, 1H)	8.28 (s, 1H)	8.32 (s, 1H)
H2	6.07 (s, 1H)	6.02 (s, 1H)	6.25 (s, 1H)
H3	7.96 (d, J = 7.7, 1H)	7.75 (s, 1H)	8.72 (m, 1H)
H4	7.17 (t, J = 7.7, 1H)	-	-
H5	7.55 (t, J = 7.7, 1H)	7.35 (d, J = 8.1, 1H)	8.72 (m, 1H)
H6	7.11 (d, J = 8.1, 1H)	7.00 (d, J = 8.2, 1H)	7.37 (d, J = 8.8, 1H)
H7	8.23–8.27 (m, 1H)	8.23–8.27 (m, 1H)	8.26–8.30 (m, 1H)
H8	8.55–8.62 (m, 1H)	8.58–8.62 (m, 1H)	8.40–8.43 (m, 1H)
H9	8.34–8.40 (m, 1H)	8.34–8.40 (m, 1H)	8.28–8.33 (m, 1H)
H10	8.26–8.31 (m, 1H)	8.26–8.31 (m, 1H)	8.29–8.35 (m, 1H)
H11	8.06–8.12 (m, 1H)	8.06–8.12 (m, 1H)	8.13–8.19 (m, 1H)
H12	8.05–8.15 (m, 1H)	8.10–8.16 (m, 1H)	8.08–8.12 (m, 1H)
H13	8.06–8.12 (m, 1H)	8.06–8.12 (m, 1H)	8.13–8.19 (m, 1H)
H14	8.26–8.31 (m, 1H)	8.26–8.31 (m, 1H)	8.29–8.35 (m, 1H)
H15	8.34–8.40 (m, 1H)	8.34–8.40 (m, 1H)	8.28–8.33 (m, 1H)
H16	–	2.33 (s, 3H)	–

* J values are reported only for well-resolved signals; overlapped aromatic resonances are reported as multiplets.

**Table 4:** Substituent-dependent absorption behavior for PC1–PC3.

Compounds	Substituent	Absorption λ_max_ (nm)	Spectral Effect	Optic Band Gap (E_g_)
PC1	–H	432	Basic π–π *	2.87
PC2	–CH_3_	433	Slightly bathochromic	2.86
PC3	–NO_2_	440	Prominent bathochromic	2.82

* excited state.

**Table 5:** Photoluminescence data of synthesized compounds.

Compounds	Absorption (nm)	Emission (nm)	Emission Intensity (cps)	Stokes Shifts (nm)
PC-1	432	540	1.17 × 10^6^	108
PC-2	433	540	1.09 × 10^6^	107
PC-3	440	565	5.59 × 10^5^	125

**Table 6:** Absorption and emission properties of selected pyrene-based Schiff bases reported in the literature.

Structure of Schiff Bases	Absorption (nm)	Emission (nm)	Properties	References
** 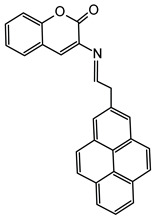 **	328, 343, 405	458	Fluorometric probe and biological application	[48]
** 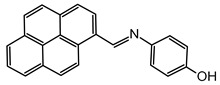 **	394	440	Photophysics and antimicrobial properties	[24]
** 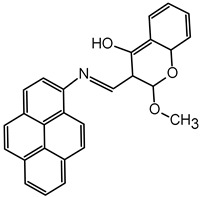 **	430	443	Detection of sarin gas mimic, diethylchlorophosphate	[41]
** 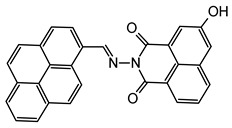 **	392	549	Fluorescent pigment	[29]
** 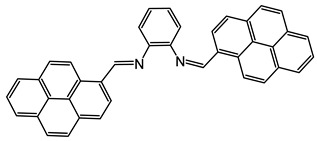 **	355	410, 428	Cell imaging and test strip	[49]
** 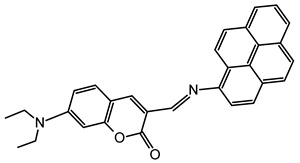 **	281, 358, 430	435	Cu-selective fluorescent sensing	[50]
** 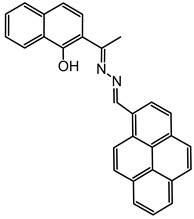 **	365	460	Fluorescence chemosensor for the detection of Cu^2+^ ions	[51]
Current study (PC-1, PC-2, and PC-3)	432–440	540–565	Photophysical properties	

## Data Availability

The original contributions presented in this study are included in the article/Supplementary Material. Further inquiries can be directed to the corresponding author.

## Supplementary Materials

The following supporting information can be downloaded at: https://www.mdpi.com/article/10.3390/molecules31061059/s1.

## Abbreviations

The following abbreviations are used in this manuscript:

OLEDs	organic light-emitting diodes
OFETs	organic field-effect transistors
DSSCs	dye-sensitized solar cells
HOMO	highest occupied molecular orbital
LUMO	lowest unoccupied molecular orbital
