# Supplementary material for: Pyrene-Chromone Schiff Base Molecules with Tunable Fluorescence: Structure–Property Relationships and Substituent Effects
Source: Molecules. 2026 Mar 23;31(6):1059. doi: 10.3390/molecules31061059 (PMC13029271; doi:10.3390/molecules31061059)
Supplement: Supplementary file 1 [file molecules-31-01059-s001.zip › molecules-4173054-supplementary.pdf]

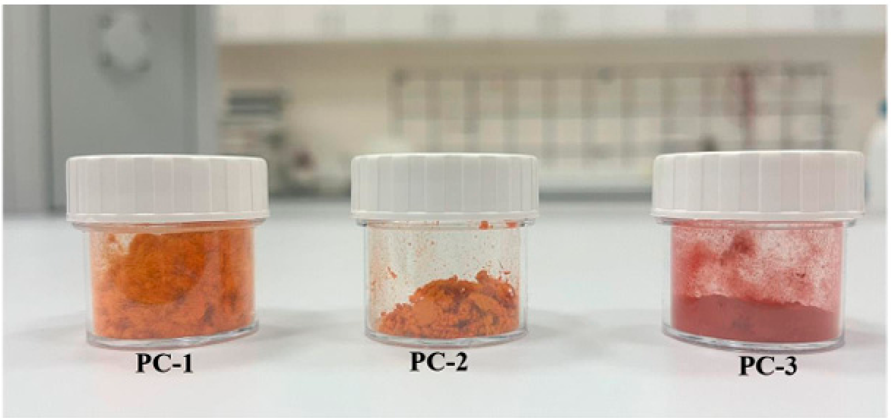

Images S1. Images of the synthesized Schiff bases in powder form.

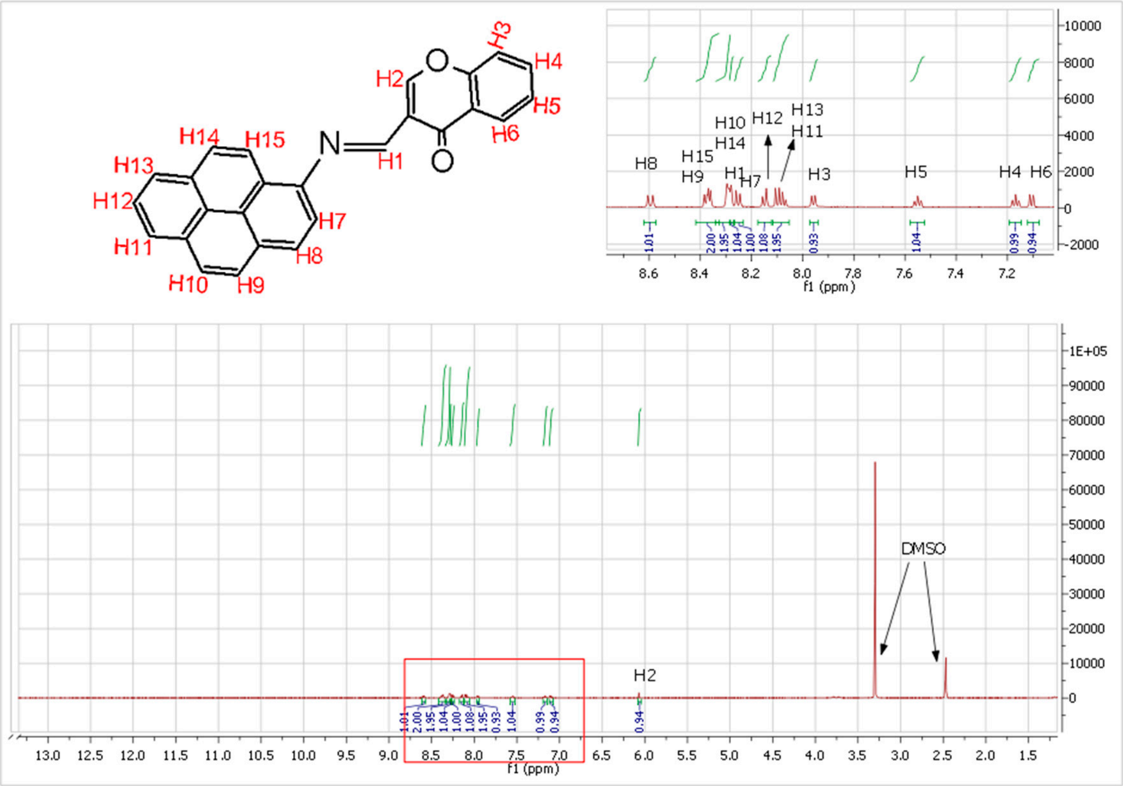

a)

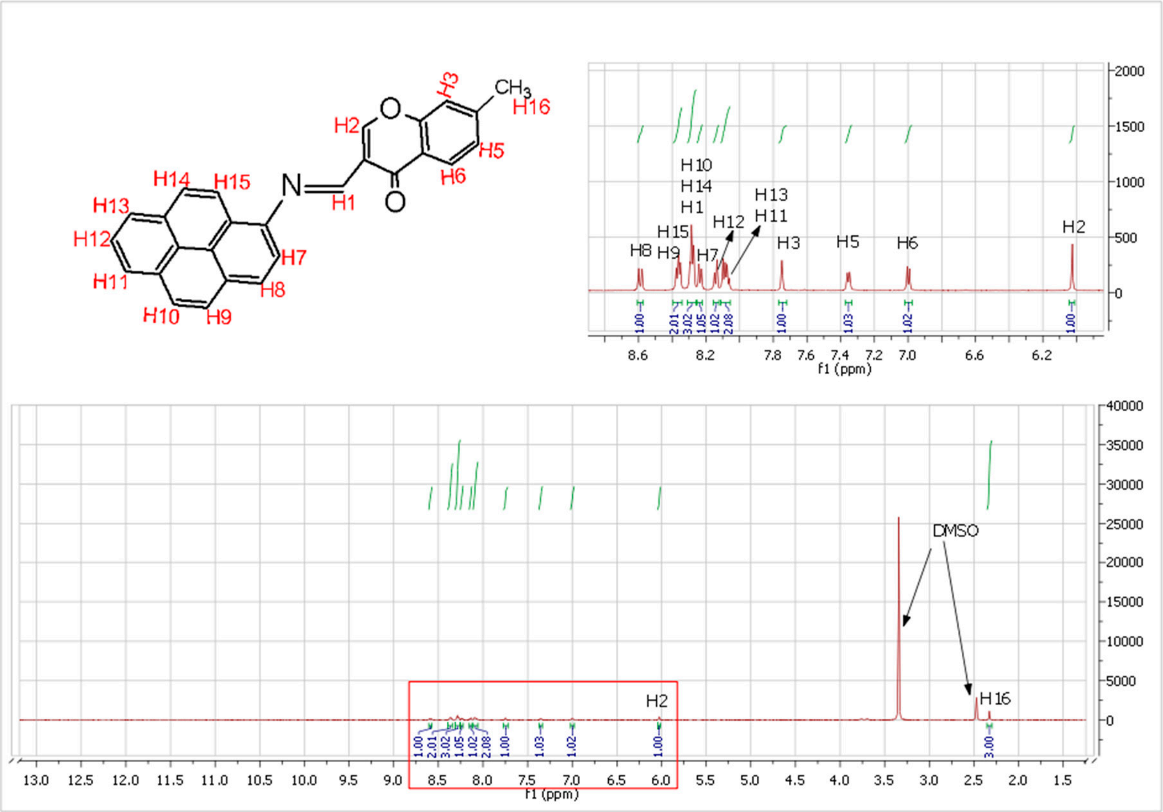

b)

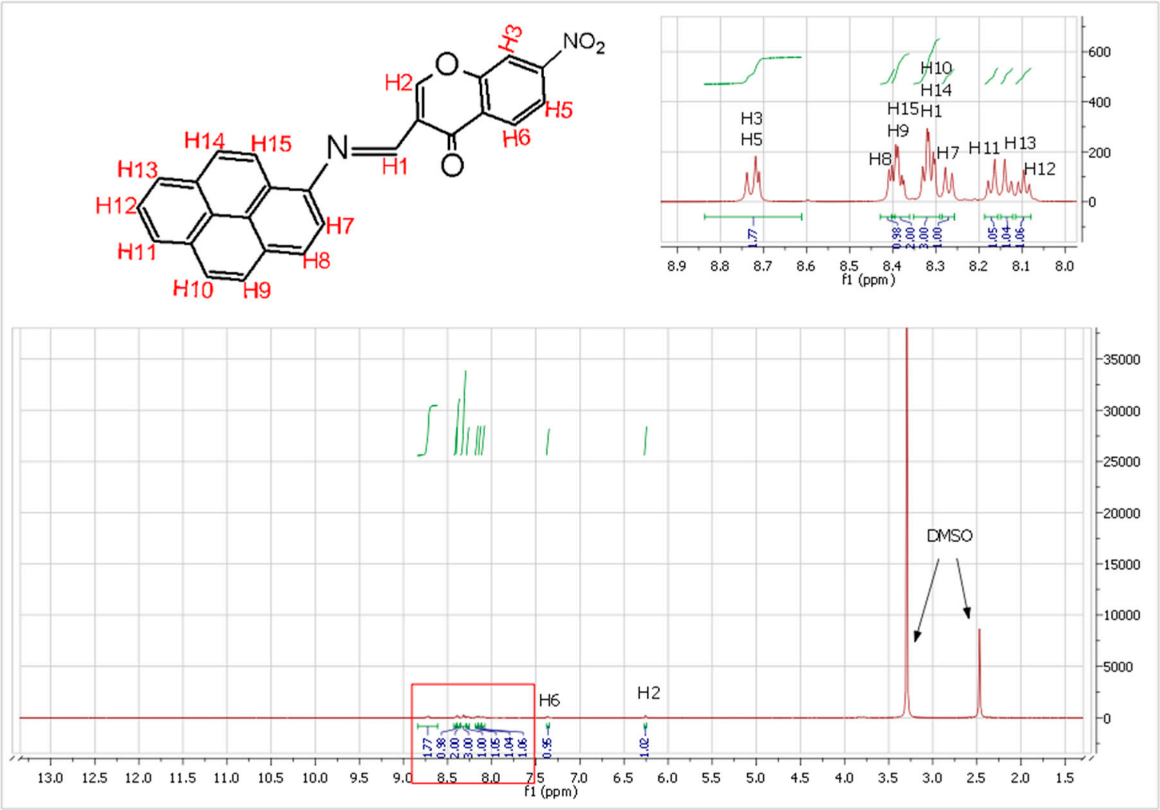

c)

Figure S1. ^{1}H-NMR spectra of a) PC-1, b) PC-2 and c) PC-3

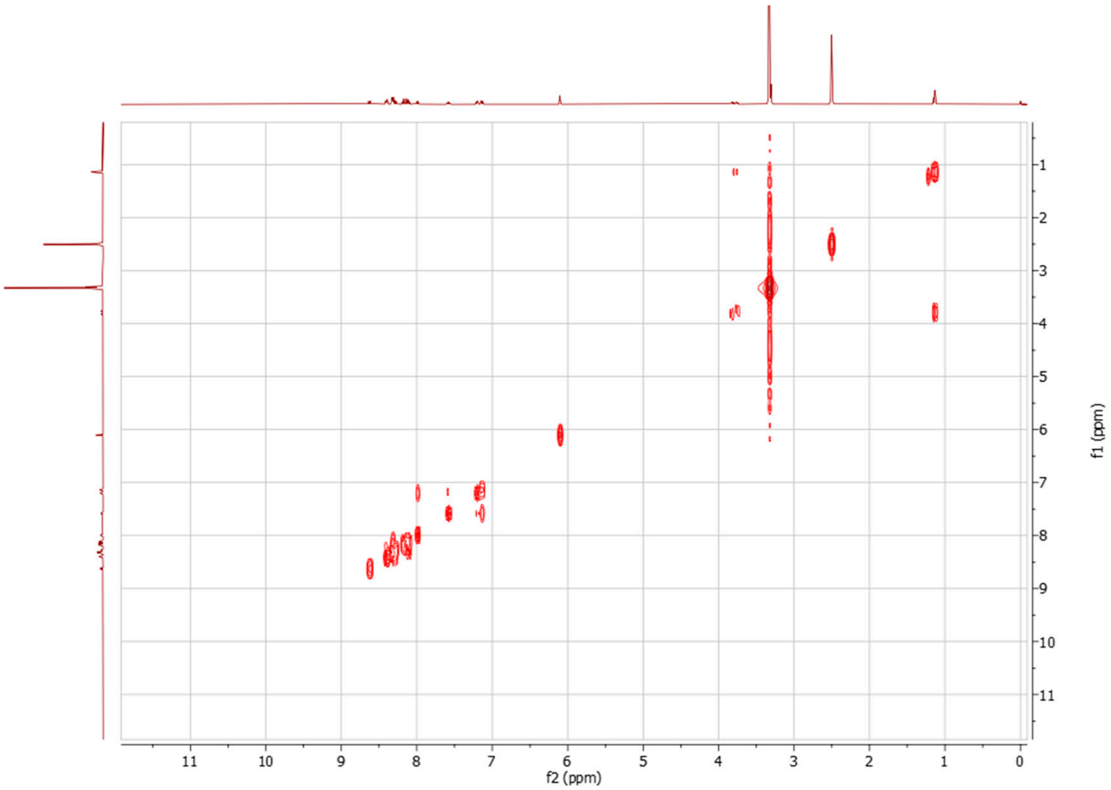

Figure S2. 2D-COSY NMR spectra of PC-1

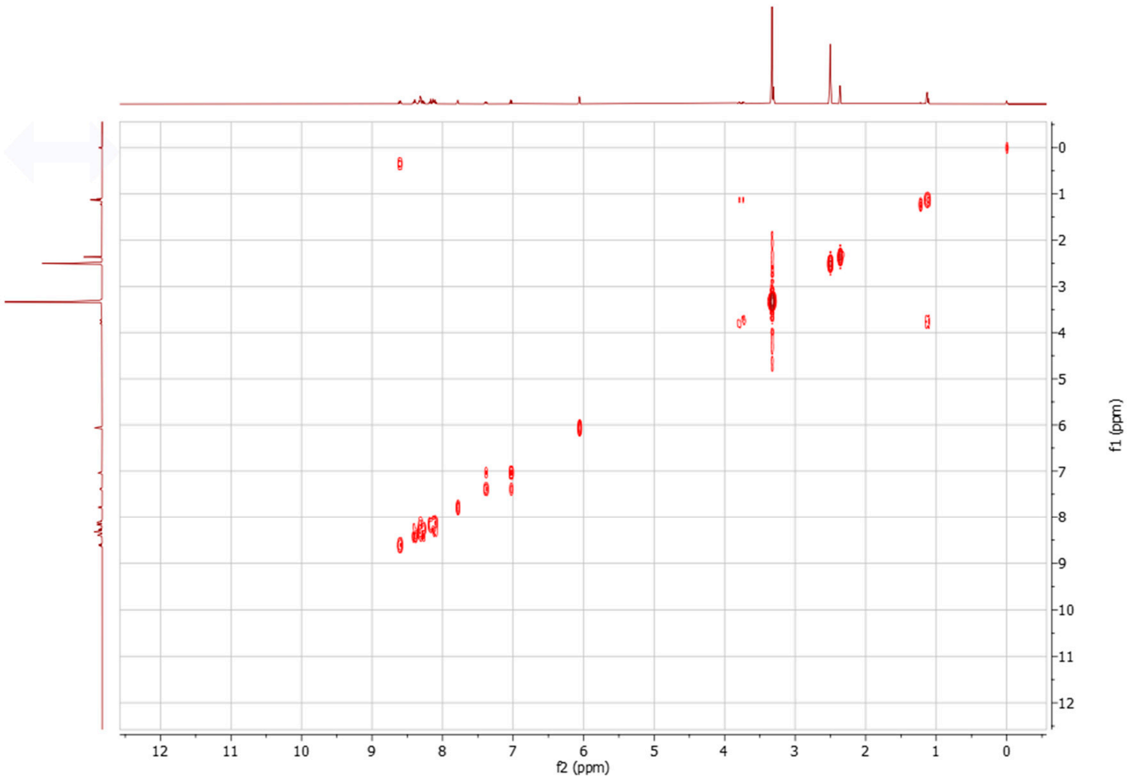

Figure S3. 2D-COSY NMR spectra of PC-2

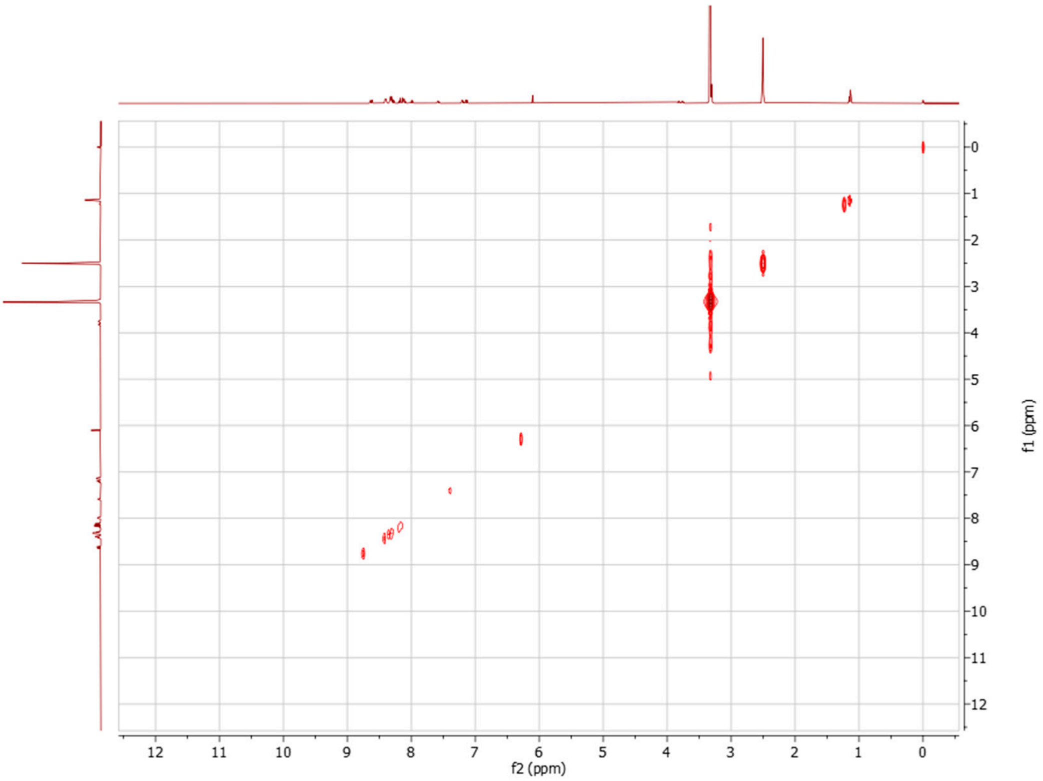

Figure S4. 2D-COSY NMR spectra of PC-3
